# Ketamine Addiction With Multisystem Toxicity: A Case Report From Canon Lan Detox Unit, Neath Port Talbot Hospital, South Wales

**DOI:** 10.1192/bjo.2026.11873

**Published:** 2026-06-30

**Authors:** Oluwafunmilayo Omisakin, Mohan Gangineni, Aled Davies

**Affiliations:** Swansea Bay UHB, Swansea, United Kingdom

## Abstract

**Aims:**

Ketamine misuse is an emerging public health concern, with increasing reports of severe physical and psychological complications. Chronic use is associated with hepatobiliary and urinary tract injury, often linked to ketamine’s metabolic pathways and toxicological effects (1-3). This report describes the complications and treatment outcomes of a young adult with severe ketamine dependence admitted to a specialist detoxification unit in South Wales.

**Methods:**

A 25 year old British male with severe ketamine dependence (up to 8 g/day) presented with abdominal pain, urinary frequency, jaundice, and weight loss. Initial investigations revealed markedly deranged liver enzymes and raised creatinine. He was diagnosed with ketamine induced cholangiopathy (2) and cystitis, conditions well documented among chronic abusers (3). Despite initial improvement during detoxification, he relapsed shortly after discharge, escalating use to approximately 90 g and requiring ICU admission for ketamine overdose with emerging multiorgan failure. After medical stabilisation, he was readmitted for detoxification and demonstrated improved motivation and abstinence, with plans for residential rehabilitation.

Management followed general principles of substance-dependence treatment due to the absence of ketamine-specific guidelines (4). Oxazepam was used to manage withdrawal due to hepatic impairment, alongside analgesia for bladder pain. Mirtazapine was prescribed for mood symptoms, and the patient engaged in relapse-prevention therapy and occupational support.

**Results:**

Ketamine addiction has risen significantly across the UK, with documented multisystem toxicity including hepatobiliary injury, bile duct fibrosis, and cholangiopathy *(1-3)*. Renal and lower urinary tract damage are similarly common in chronic users *(3)*. This case demonstrates how rapid deterioration and multiorgan failure can occur following relapse, underscoring the need for sustained support after detoxification.

**Conclusion:**

Ketamine abuse can cause severe and potentially irreversible liver and kidney damage. Relapse following detoxification carriesrisk of multiorgan failure. Sustained abstinence and structured follow-up are essential.

